# Assessing public health preparedness and response in the European Union- a review of regional simulation exercises and after action reviews

**DOI:** 10.1186/s12992-023-00977-y

**Published:** 2023-10-28

**Authors:** Mari Nythun Utheim, Mohamed Gawad, Karin Nygård, Emily Macdonald, Monica Falk

**Affiliations:** Norwegian Institute of Public Health, Postboks 222 Skøyen, Oslo, 0213 Norway

**Keywords:** IHR, Monitoring and evaluation, Simulation exercises, After action review, Public health preparedness, European Union

## Abstract

**Background:**

Improving response capacities in the EU requires a good overview of capacities at both country and Union level. The International Health Regulations (2005) Monitoring and Evaluation framework assesses capacities in countries. It includes semi-quantitative tools such as State Parties Annual Report (SPAR) and Joint External Evaluation (JEE). After Action Reviews (AAR) and Simulation Exercises (SimEx) were included to identify weaknesses in the functionality of capacities which are not addressed bySPAR and JEE. This study presents an analysis of the use of qualitative tools at regional level, in Europe. It aims to identify their added value by comparing them to standardised monitoring tools and lessons learned from COVID-19, and considers ways to improve their use in assessing capacities in the EU.

**Methods:**

We included 17 SimEx and 2 AAR organised by the European Commission between 2005 and 2018. We categorised a total of 357 recommendations according to the IHR (2005) core capacities and to the target audience of the recommendation. We analysed the data using language analysis software. Recommendations to countries were compared to SPAR and JEE indicators. Recommendations to EU agencies were compared to the current mandates of the EU agencies, and to lessons learnt during COVID-19.

**Results:**

Of all extracted recommendations from the exercises, 59% (211/357) targeted EU agencies, 18% (64/357) targeted countries, and 16% (57/357) targeted both. Recommendations mainly addressed areas of IHR coordination (C2), heath emergency management (C7) and risk communication (C10), and not low scoring areas. Recommendations complement SPAR indicators by identifying gaps in functionality. Eight out of ten early lessons learnt during the COVID-19 pandemic had been raised earlier as recommendations from exercises. Exercise reports did not include or result in action plans for implementation, but COVID-19 has accelerated implementation of some recommendations.

**Conclusion:**

SimEx/AAR provide valuable insight into public health preparedness at EU level, as they assess functionality of preparedness and response mechanisms, point out gaps, and provide training and awareness on for participants, who often have key roles in public health emergencies. Better follow-up and implementation of recommendations is key to improve the regional preparedness for international public health incidents such as pandemics.

**Supplementary Information:**

The online version contains supplementary material available at 10.1186/s12992-023-00977-y.

## Background

The threat of emerging diseases to global health security has increased due to human behaviour, population growth, globalisation, global environmental changes and other factors [1]. Nation states remain the most important actors to improve global health security.

The International Health Regulations 2005 (IHR 2005) monitoring and evaluation framework enables countries to assess their IHR (2005) core capacities. It has four components: State Party self-assessment annual reporting (SPAR), Joint External Evaluation (JEE), After Action Reviews (AAR), and Simulation Exercises (SimEx). The SPAR and JEE are semi-quantitative monitoring tools with standardized indicators which track capacities over time and allow for comparison. However, these indicators may not adequately reflect the functionality of existing preparedness systems and capacities, and can obscure important gaps [2]. For instance, both frameworks were criticized for their inability to predict national needs in the response to the COVID-19 pandemic [3, 4].

AAR and SimEx were included in the monitoring and evaluation framework as these tools may better expose weaknesses in the functionality of preparedness capacities. SimEx and AAR have also been used at the European level, involving EU agencies and multiple countries. In addition, a sub-type of AAR, Intra-Action Reviews (IAR), have been carried out in events of longer duration to guide an ongoing response. However, the results from AAR and SimEx are not standardized and may be difficult to link to specific preparedness capacities or indicators.

In October 2022, the EU adopted the final building blocks of the European Health Union, where strengthening health preparedness in the Union is central. Significant developments have been made with the updated Regulation on Serious cross-border health threats, the extended mandate of the European Centre for Disease Prevention and Control (ECDC) and the Emergency Framework Regulation which extends the powers of the European Health Emergency Preparedness and Response Authority (HERA). In order to improve preparedness and crisis response in the EU, a comprehensive overview of strengths and weaknesses at both country and Union level is needed.

This study assesses the feasibility of using qualitative tools as part of the monitoring and evaluation of regional public health preparedness and response. The intended outcome is improved use of results from SimEx and AAR as part of a comprehensive monitoring and evaluation strategy for IHR (2005) core capacities and implementation of cross border health threats regulation at the European level.

The following research questions are addressed:

- What is the added value of qualitative tools (SimEx and AAR) in the IHR (2005) monitoring and evaluation framework?
- What are the main recommendations resulting from these qualitative tools at the EU-level?
- How can the use of these tools be improved to maximize their added value in improving preparedness in the European context?
- To what extent do recommendations from SimEx and AAR conducted before the pandemic correspond to lessons learned in the EU response to COVID-19?

## Methods

### Data collection

We retrieved a list of SimEx and AAR conducted by the European Commission from the database maintained by DG SANTE in CircaBC. Reports from each exercise were accessed from the database or found by web search. For each exercise, we extracted information about the organisation of the exercise (number of MS and EU agencies involved, duration), the scope (objective, hazard and cause) and results (recommendations and target audience).

We obtained data on SPAR and JEE scores from the WHO e-SPAR platform [5] and the Strategic Partnership for Health Security and Emergency Preparedness (SPH) Portal [6] respectively. SPAR scores for the EU, EEA and Switzerland were calculated using the average score for all countries that had responded from the period of 2010–2018 (*n* = 32). JEE scores were calculated using all available JEE reports for EU/EEA Member States and Switzerland from 2010 to 2018 (*n* = 9). The SPAR framework went through updates in 2017, 2018 and 2021, whereby some capacities were merged, renamed, or added. In order to be able to compare scores over the years, we adapted and aligned the SPAR core capacity framework from pre-2021 to the current framework.

### Sample

We included SimEx/AAR that involved at least one EU agency in addition to EU/EEA states and Switzerland, and search period for inclusion was between 2005 and 2021. However, no SimEx/AAR were carried out after 2018. Workshops and trainings that did not include an exercise component were not included.

### Data categorisation

We categorized each extracted recommendation from SimEx and AAR by IHR core capacity and by indicator. Some recommendations were relevant for several core capacities, in which case the most relevant one was selected. If a recommendation could not be linked to a capacity and/or indicator, we categorised the recommendation as “Not linked to an IHR capacity”. The attribution of a core capacity to a recommendation was done by one person and then reassessed by a second person. Inconsistencies were discussed and a decision reached by consensus. Recommendations were also sorted according to their target audience (countries, regional agencies, or both). In many recommendations, the target audience was not clear. In some cases, it could be deducted from the report. In others, the target audience was left unspecified.

### Analyses

SHINY language analysis was performed to find recurrent issues/recommendations throughout the material. SHINY is a software that identifies and counts words and phrases in text [7]. Searches were done for nouns and adjectives. Relevant high frequency terms were identified and used as search terms to identify clusters of topics in the recommendations which were then described. Recommendations targeting countries, or both countries and regional agencies, were summarized according to IHR capacity and compared with the corresponding SPAR indicators for each capacity. This analysis was used to determine the added value of the exercises, and to clarify which capacities were most frequently assessed in the exercises. Recommendations to EU agencies were compiled and compared to the current mandates of EU agencies, to assess the status of implementation of recommendations. Lastly, we compared the recommendations in our dataset to lessons learnt from the COVID-19 pandemic. For this comparison, we used the communication “Drawing the early lessons from the COVID-19 pandemic” from the EU Commission [8]. Although there are many sources of lessons learned from the COVID-19 pandemic, the communication from the commission was chosen as it focuses on health preparedness and response in Europe, and provide insight in recommendations from the commission itself on where EU needs to act.

## Results

**Fig. 1:**
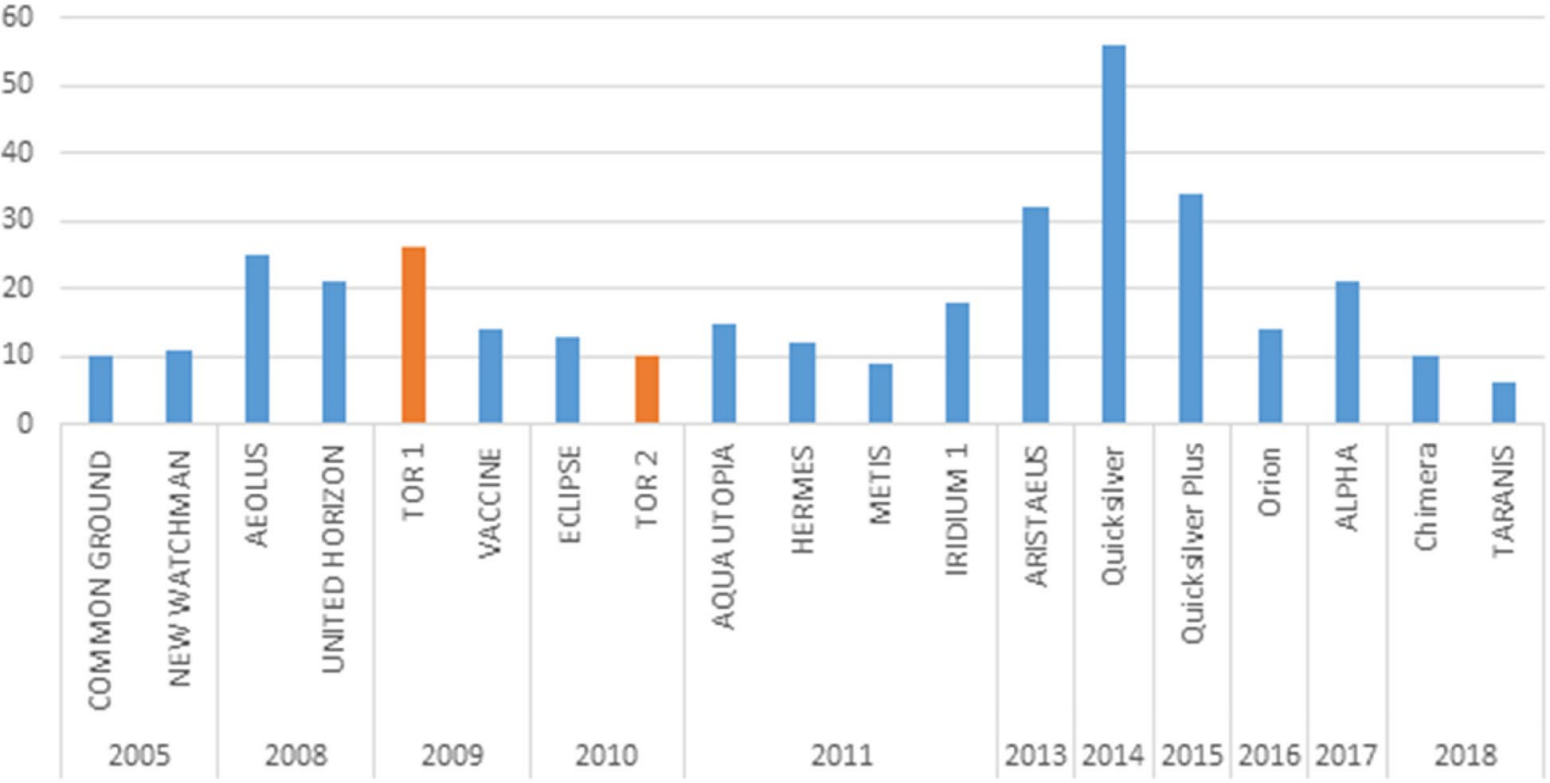
Number of recommendations per SimEx (blue) and AAR (orange) in chronological order (total *N* = 357)

In total, there were 17 SimEx and 2 AAR registered in the EC database, for which all reports were available and included in the study (Table 1). These 19 reports published between 2005 and 2018 included a total of 357 recommendations. The two AAR (subtype IAR) were carried out during the H1N1 outbreak in 2009. Each exercise provided between 5 and 55 recommendations (Fig. 1).

**Table 1:** Overview of the SimEx/AARs main objective, event and number of countries participating

Exercise	Thematic area	Event	No. of countries
NEW WATCHMAN	Communication	Contamination of a protein supplement drink	28
COMMON GROUND	Influenza preparedness	Nipah virus	28
United Horizon	Hedis tool	Contaminated illicit drug	20
AEOLUS	Information sharing	Contaminated powdered milk with Salmonella	27
TOR 1	Influenza preparedness	Deliberate release of plague/cyber attack	21
Vaccine Workshop	Vaccine	Pandemic influenza	17
ECLIPSE	Intersectoral coordination	Deliberate contamination with radioactive material	11
TOR 2	Vaccine	Monkeypox	30
IRIDIUM 1	Communication	Leakage of hydrofluoric acid on ferry	4
AQUA UTOPIA	Communication	Re-emergence of SARS-CoV	19
HERMES	Laboratory	Outbreak of smallpox	19
METIS	Laboratory	New variant of chikungunya; schistosomiasis	17
ARISTAEUS	Intersectoral coordination	Collision of chemical cargo ship; forest fire and chemical plant explosion; deliberate release of chemical	27
Quicksilver	Cross-border coordination	Unidentified illness from chemical exposure; chemical release from oil refinery explosions and freight train derailments	22
Quicksilver Plus	Cross-border coordination	Pandemic influenza/space weather event	16
Orion	Preparedness and response	Pandemic influenza	22
ALPHA	Intersectoral coordination	Pandemic influenza	20
TARANIS	Cross-border coordination	Measles-like illness in Asia	21
Chimera	Intersectoral coordination	Pandemic influenza vaccine adverse events or efficacy issues	27

Of the SimEx, twelve were discussion-based exercises or tabletop exercises (TTX) while five were command post exercises (CPX). Of the health events included in the exercise scenarios/AAR, 74% were biological events (*n* = 14), including two food safety event and one zoonotic event, 26% were chemical events (*n* = 3) or radiological events (*n* = 2). 53% of the health events of the SimEx were of natural cause (*n* = 9), and 47% of deliberate causes (*n* = 4), accidental causes (*n* = 2), or both deliberate and accidental causes (*n* = 2).

All EU countries were invited to participate in all the SimEx/AAR. EEA countries were invited to most of them (16/19), and Switzerland to over half (11/19). In all the exercises, public health specialists, including the Health Security Committee (HSC) members, were in the participant target group. For 42% of the SimEx/AAR (*n* = 8), at least one other target group was specifically invited to participate, including communications specialists (members of the HSC COMNET) (*n* = 5), chemical sector (*n* = 2), environmental health (*n* = 2), food safety (*n* = 2), veterinary health (*n* = 1), civil protection/security (*n* = 1), transportation (*n* = 1) and energy (*n* = 1). For the CPX, participating countries were able to invite other relevant sectors. The most represented international or EU agencies were WHO Euro and/or HQ (*n* = 15), DG SANCO/DG SANTE (*n* = 15), ECDC (*n* = 13), CHAFEA (*n* = 5), DG HOME (*n* = 4), JRC (*n* = 4), DG ECHO (*n* = 4), EMEA (3) and EFSA (*n* = 3).

### Classification of recommendations

**Fig. 2:**
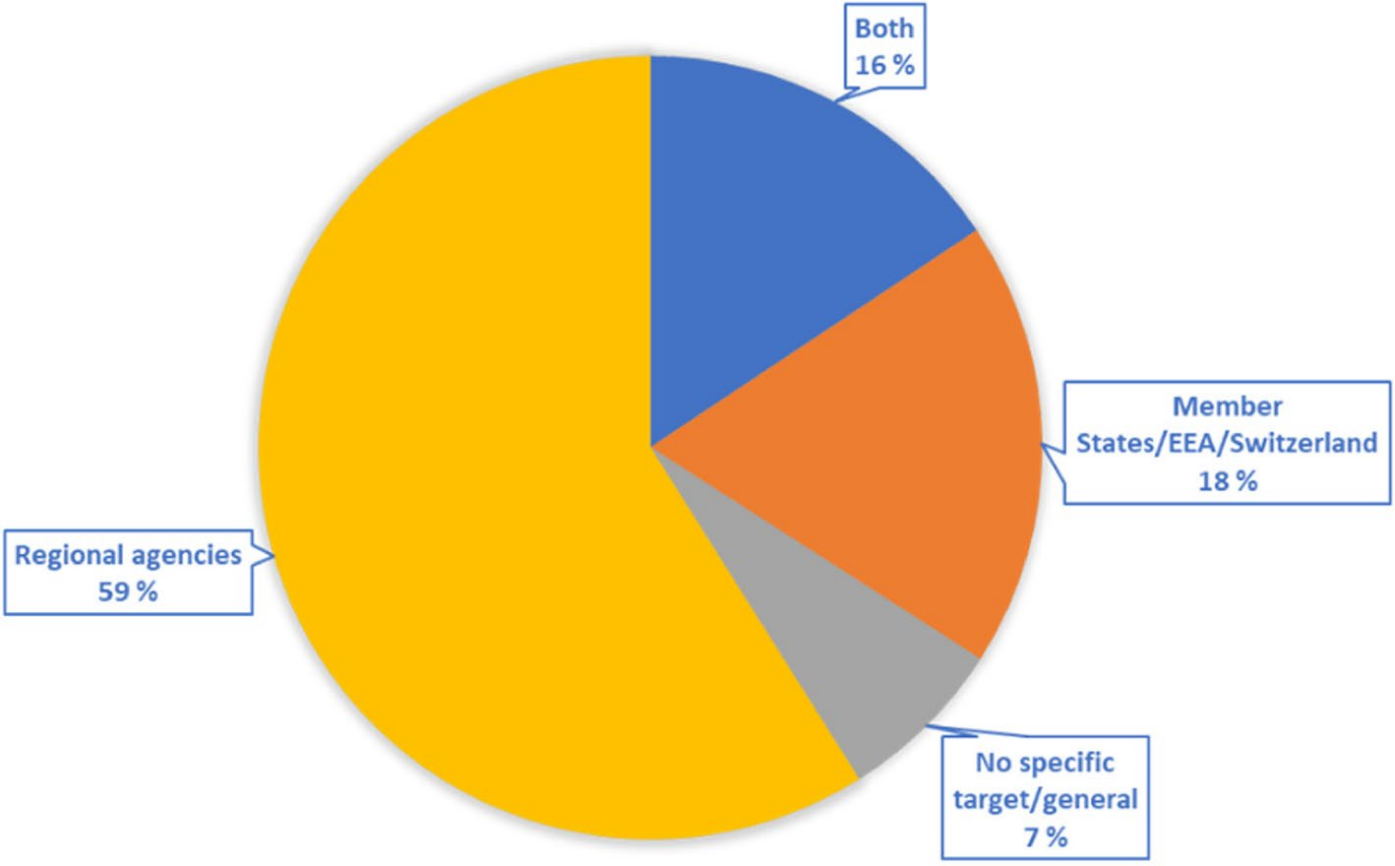
Recommendations per responsible entity, as a proportion (*N* = 357)

Recommendations from the exercises were targeting either EU agencies, Member States/EEA/Switzerland, or both. Figure 2 shows the proportion of recommendations to EU agencies, Member States/EEA/Switzerland, both, or none specified.

In many exercises it was difficult to determine who was responsible for following up recommendations. Out of the 19 exercise reports, only 6 stated the agency or organisation responsible for following up each recommendation. In other reports, only some of the recommendations were addressed to a particular entity.

**Fig. 3:**
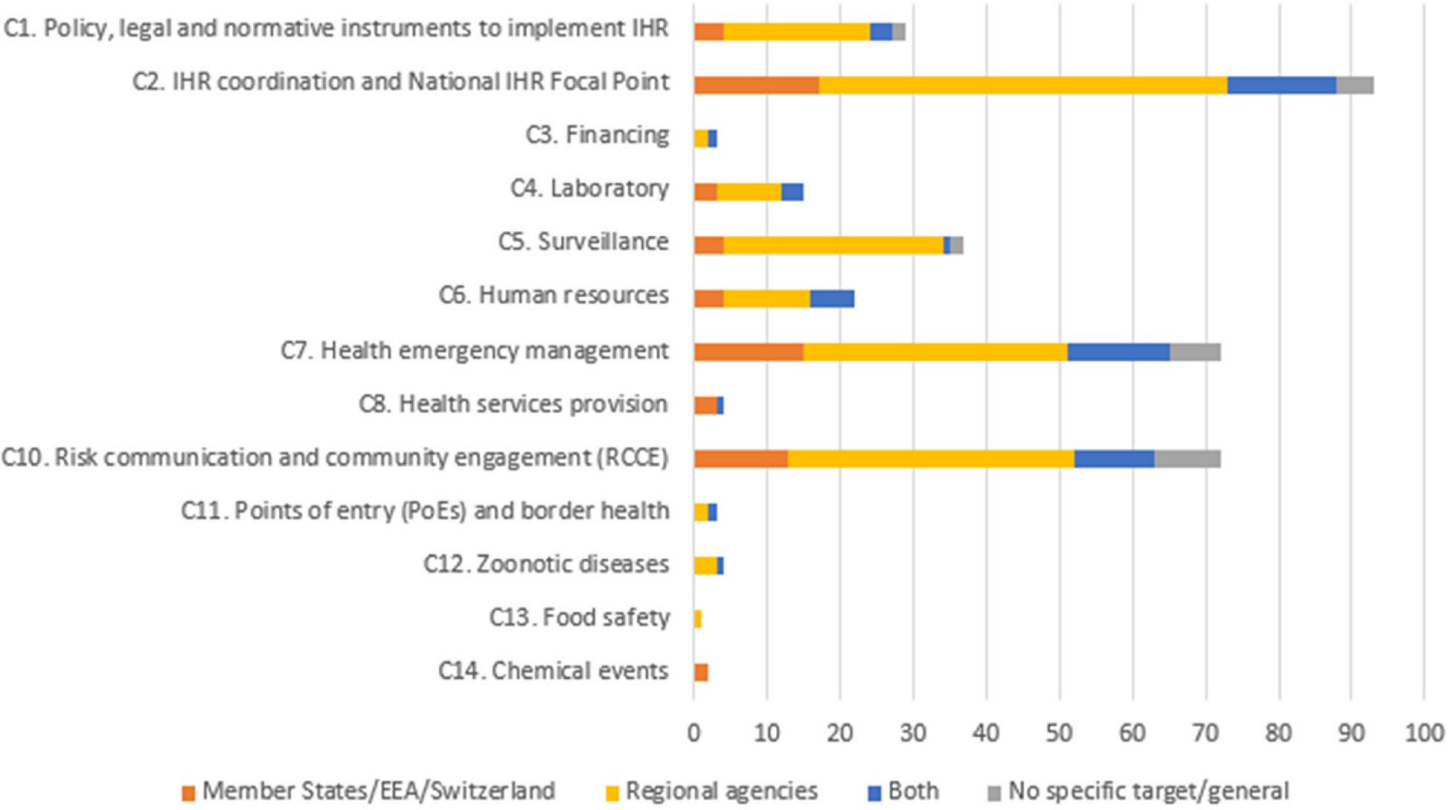
Number of recommendations per IHR capacity, by target audience (*N* = 357)

Classifying all the recommendations individually by IHR capacity gave an overview of which capacities were most frequently addressed in the exercises. There were recommendations concerning all capacities except C15: Radiation emergencies (Fig. 3). The most recurrent recommendations in this dataset were concerning C2 IHR coordination (26%), C7 Health emergency management (10%) and C10 Risk communications (10%). Least recurrent were recommendations concerning the capacities C13: Food safety (< 1%) and C14: Chemical events (1%).

### Most common recommendations

Across core capacities, the most common topics were the following:

- *Collection, exchange and sharing of information*: 34 out of 357 recommendations stipulated the importance of regular information flow and improving ways of information exchange. Information flow across borders was especially underscored as an essential element in managing events, with another 27 out of 357 recommendations concern the role and use of Early Warning and Response System (EWRS). Intersectoral communication at the national level was also mentioned repeatedly. Several recommendations concerned risk communication, including interaction between authorities and media and social media, and how to ensure transparent, coherent, scientifically correct, and timely information to the public.
- *Systems, procedures, and protocols (SOPs):* Thirty four recommendations across six different capacities highlighted a need to streamline and standardise procedures to facilitate international collaboration during public health events.
- *Clarification and awareness of roles:* A recurrent issue across capacities was the review, clarification and awareness of roles and responsibilities of different entities, particularly EU entities. In total 33 out of 357 recommendations related to this topic, of which 29 specified the need to clarify roles of EU agencies, most often the EU commission itself or DG SANTE. The remaining 4 recommend clarification of roles on national level within countries.

A recurrent statement in the summary of the execution of the exercise was that participants felt that the exercise itself was useful and that it allowed them to practise skills and identify gaps in preparedness and response.

### Recommendations to EU/EEA Member States and Switzerland

34% of the recommendations targeted countries, either exclusively or in addition to regional agencies. They focused on the same capacities as the overall recommendations, as seen in Fig. 3. These were compared with SPAR indicator for the corresponding capacity, to assess whether and how they add value to the SPAR framework. The comparison is detailed in Additional file 1: Annex 1. For most of the capacities, the recommendations provided added value in the form of identification of specific gaps, either in terms of a particular type of event or disease. Numerous recommendations also pointed out that despite the implementation of a tool, role or mechanism, there are weaknesses such as lack of awareness or incorrect use, which indicated that despite the existence of required technical or infrastructural capacities, they were not considered to be functioning optimally.

### Recommendations to EU agencies

The recommendations are summarized in Additional file 1: Annex 2. *The improvement of information exchange tools* was mentioned in 13 out of the 19 reports assessed. The need to upgrade, improve and integrate information exchange tools, as well as to train users was mentioned in various exercises. There were recommendations on the need to streamline and create integrated platforms for sharing information during public health crises. *Emergency coordination* was another recurrent topic addressed in 13 out of 19 reports. Recommendations pertained both to roles and responsibilities, as well as to multisectoral coordination depending on the type of event. There was a recurrent call for a clearer mandate for coordination. A series of recommendations on how to strengthen the HSC specifically, and HSC COMNET, came in 2011. There were also several recommendations on *joint procurement and regional stockpiling* of medical countermeasures. *Streamlining public communication,* both between countries and regional entities, was another frequent recommendation. This included how to engage on social media. Some recommendations were specific to the EU level coordination and intersectoral collaboration on *hybrid and chemical events*.

### COVID-19 lessons learnt

A step-by-step comparison of the 10 lessons learnt from COVID-19 from the EU Commission’s 2021 report with the SimEx recommendations found that many challenges faced in Europe during the 2020–2022 pandemic had been identified in exercises dating as far back as 2005 and as recently as 2018. Table 2 outlines eight of the COVID-19 lessons learnt for which similar recommendations have been found in earlier SimEx/AAR. For the two lessons not presented, there were no obvious similarities. A more detailed comparison can be found in Additional file 1: Annex 3.

**Table 2:** Comparison between lessons learnt during Covid-19 and recommendations in exercises

Covid-19 lesson learnt	Similar recommendation found in (name of SimEx/AAR)	Year of exercise
Lesson 1*:**** “Faster detection and response depends on stronger global surveillance and more comparable and complete data.”***	COMMON GROUND Influenza preparedness	2005
	TOR 1 Influenza preparedness	2009
	ARISTAEUS biological threat (food safety)	2013
	ALPHA biological threat (zoonotic)	2017
	TARANIS biological threat (pandemic flu)	2018
Lesson 2: ***“Clear and coordinated scientific advice facilitates policy decisions and public communication.”***	IRIDIUM Chemical event	2011
	Quicksilver Chemical event	2014
	Orion Biological treat (chikungunya outbreak)	2016
Lesson 3: ***“Preparedness needs constant investment, scrutiny and review”***	TOR 1 Influenza preparedness	2009
	Quicksilver	2014
	Quicksilver Plus	2015
	Chimera Chemical event/Hybrid threat	2018
Lesson 5: ***“Coordinated measures should become a reflex for Europe”***	COMMON GROUND Influenza preparedness	2005
	TOR1 Influenza preparedness	2009
	Quicksilver Plus chemical event	2015
	TARANIS biological threat (pandemic flu)	2018
Lesson 6: ***“Reinforced public–private partnerships and stronger supply chains are needed for critical equipment and medicines”***	COMMON GROUND Influenza preparedness	2005
	TOR1 Influenza preparedness	2009
	TOR 2 influenza vaccination	2010
	TARANIS biological threat (pandemic flu)	2018
Lesson 7: ***“A pan-European approach is essential to make clinical research faster, broader and more effective”***	TOR 2 influenza vaccination	2010
Lesson 8: ***“Capacity to cope in a pandemic depends on continuous and increased investment in health systems”***	TOR 1 Influenza preparedness	2009
	TARANIS biological threat (pandemic flu)	2018
Lesson 10: ***“A more coordinated and sophisticated approach to tackling misinformation and disinformation should be developed”***	VACCINE WORKSHOP influenza vaccination	2009
	TOR 1 Influenza preparedness	2009
	TOR 2 influenza vaccination	2010
	ALPHA biological threat (zoonotic)	2017

## Discussion

Recurrent recommendations from the SimEx/AAR on EU level can be roughly organised in three areas.

1. Improve information sharing between sectors, countries and between EU agencies and country level. This concerned both SOPs for information sharing and communication tools.
2. Aachieve a certain standardisation of procedures across sectors and countries on how to respond to incidents of varying severity and extent.
3. Improve clarity and awareness of the roles of different EU agencies in crisis management.

About a third of recommendations targeted Member States, EEA countries and Switzerland. Two thirds targeted EU agencies exclusively. They did not focus particularly on low scoring capacities as measured in the IHR SPAR tool. The most addressed IHR core capacities were C2 IHR coordination, C7 Health Emergency Management and C10 Risk communication. This is in line with the WHO review of national level SimEx and AAR globally conducted between 2016 and 2019 [9], which hypothesises that cross-cutting capacities are more likely to be in the foreground of the regional response in a real-life event. We can make the same hypothesis in this review. As some capacities lend themselves more to be trained in an exercise, recommendations will also more frequently address these. Further, the exercises included in our analysis had international, intersectional and EU agency representation, which would equally facilitate evaluation of capacities that involve coordinated actions from several entities and sectors.

SimEx and AAR have several types of added value in the in the IHRMEF. First, preparedness and response at the *regional* level is not covered by any other part of the IHRMEF. Secondly, it is important to distinguish between “monitoring” and “evaluation” when comparing quantitative tools (SPAR/ JEE) and qualitative tools (SimEx/AAR). *Monitoring* implies regular data collection to assess progress against a defined target. In the IHRMEF, the SPAR annual reporting constitutes monitoring—its regularity and completeness of reporting enables mapping of structures in place and the continuous assessment of the development of capacities over time. Evaluation assesses the overall functionality and response time. SimEx and AAR constitute context-specific evaluations of the functionality and effectiveness of the structures in place, thus complementing SPAR and JEE. And finally, SimEx and AAR identify important gaps in public health preparedness, demonstrated by comparison with challenges experienced during COVID-19.

We found that eight out of ten of the lessons learned from COVID-19 in the EU Commission’s 2021 report had already been identified in our SimEx/AAR, and action points had been proposed. This indicates on the one hand that the qualitative tools assessed here are topically relevant and pertinent and identify important gaps in the national and international response. An analysis from 2019 reviewing the effectiveness of AAR as a tool supports this finding [10] and concludes that AARs hold considerable promise as a tool to improve public health preparedness and organizational learning.

One the other hand, it reveals a problem with follow-up and implementation. For example, recommendations to EU agencies focused on improving reliability of information exchange and coherence of public communication, increasing regional emergency coordination and achieving more regional solutions to providing medical countermeasures. These challenges persisted in the COVID-19 pandemic. Both the EU Commission’s 2021 report and the ECDCs technical report [11] focus on international cooperation and coordination, and the need for more coordination of messages at EU level as areas for improvement. Joint procurement and stockpiling of medical countermeasures are the main topic addressed in lesson 6 from the commission. Another example is the Commissions lesson 1, highlighting weaknesses in the international alert and surveillance system. This echoes recommendations from five previous SimEx and AAR. As early as 2005, in the SimEx “Common Ground”, it was recommended to establish efficient on-line, real-time data input in a crisis situation, accessible for relevant bodies. Similar recommendations came in 2009, 2013, 2017 and lastly in 2018, when strengthened information sharing and surveillance between EU agencies and national and international partners were underlined in the pandemic flu SimEx “Taranis”. Shortages of healthcare staff and the need for surge capacity are important issues raised in the ECDC report and the Commission’s. This has been pointed out in earlier recommendations underlining that frontline and support staff shortage in a pandemic situation would severely hamper capability and capacity. Lastly, combating misinformation and disinformation, lesson 10 from the Commission, has been repeated in recommendations over the years raising the issue on how to counter the “anti- vaccine lobby”, monitor accuracy of public health messages, and develop a vaccination strategy during a pandemic. These similarities between exercise recommendations and COVID-19 lessons learned indicate that these gaps have persisted over a number of years and remain a challenge in the regional preparedness and response capacity. Could a better implementation of recommendations throughout the years have strengthened the early response to COVID-19 in Europe?

Indeed, one of the main challenges with SimEx and AAR is follow-up and implementation of recommendations. We did not find consistent assignment of responsibility to implement the recommendations. The exercises and resulting reports were commissioned from exercise management teams, who do not have the authority to develop action plans for implementation of the recommendations. There is frequent use of passive language, and some recommendations are too general or vague for it to be possible to assign responsibility for follow up or measure implementation progress. As a result, the output and impact of the exercises themselves are difficult to measure and likely reduced. This finding is consistent with other literature. A review of AAR reports from anthrax bioterror attacks, SARS outbreak, 2009 H1N1 pandemic, and West African Ebola epidemic reveals a similar pattern of repeated weakness and failure [12]. The phenomena are here described as “lesson observed but not lesson learned”. During the AAR/SimEx global consultation in 2019, the implementation of AAR & SimEx recommendations and findings was highlighted as an area that “remains challenging” [13]. One of the outcomes of the consultation was thus a recommendation to draft “an implementation framework integrating recommendations and action plan into operational planning that should be shared with the Member States to enhance accountability and national ownership and develop a 1–2-page strategy for the implementation of AAR & SimEx finding”. This was also one of the main conclusions in the WHO report on Covid-19 Intra Action Reviews, where one recommendation was to “identify a reliable and systematic approach to monitor AAR recommendations to ensure they progress within the proposed timeline and meet the desired outcomes” [14].

There are some initiatives underway to address the challenges associated with following up recommendations from SimEx and AAR. In 2018, the WHO issued a guideline for implementing SimEx and AAR. The guidelines include a detailed guide on how to develop recommendations which are “specific, feasible, time bound, measurable, and adequately translated into an action plan” [15]. Another WHO guideline for COVID-19 IAR from 2020 [16] underline that implementation of proposed activities should be monitored by a designated follow-up team. All the SimEx/AAR in this report are from 2018 or before, but it will be important that organisers of exercises use these manuals in the future. Recent COVID-19 IAR at EU level and in Ireland are examples that shows this guideline is in use [17–19]. An AAR registry is another measure proposed to facilitate organizational improvement [10]. To further embed the implementation, another measure could be that when the Commission orders a SimEx or AAR to be carried out, they have already assigned the responsibility to monitor the follow-up of recommendations to a unit in the EU with the necessary authority to develop action plans and ensure accountability. This would mitigate the fact that recommendations so far have been proposed without any authority to implement them.

Over the years, some recommendations have however been implemented, and we believe the pandemic has had a triggering or accelerating effect on many of these processes. Some relevant recent developments are the European Health Union, which aims to improve preparedness and response capacity and resilience of health care systems in the EU. A new regulation on serious cross-border threats replacing decision 1082 [20] has been adopted, which replaced the Decision on cross border health threats and provide a strengthened framework for regional coordination of health emergencies in the EU. The regulation includes “clear provisions for the EU and Member States to adopt similar and interoperable plans at national and local levels. To ensure these plans are actually operable in times of crisis, regular full-scale exercises and carry-out after-action reviews to implement corrective measures will be organised” [21]. The issue of interoperability was also addressed in part by the ECDC as a result of lessons learnt from the 2009 influenza pandemic, in the “Guide to revision of national pandemic influenza preparedness plans” [22]. Further, the establishment of HERA in 2021 was meant to ensure a coordinated approach to ensuring access to medical countermeasures during crises.

These developments in the European Health Union will be highly relevant to address the challenges with interoperability and standardisation, which were raised numerous times in our data. In a crisis, valuable time can be gained from having streamlined structures and strategies in the countries, as it facilitates collaborative efforts across borders. Further, formalising operating procedures is a way of ensuring efficient, predictable, and coherent responses during international crises. The need for clarification and awareness of roles also arises especially in times of crises when swift reactions hinge on a thorough understanding of the roles and responsibilities. However, as many of the new developments in the European region are in initial stages, it will be necessary to monitor if these efforts, in fact, lead to improvements, both at national and regional levels.

There is an increased focus on preparedness planning, assessment and reporting at national levels in Articles 6, 7 and 8 of the updated Regulation, but no specific references to how the use or follow-up of exercises and AAR should be integrated into these activities [20]. Article 5 obligates the Commission to carry out simulation exercises and after-action reviews “as required” and update a Union health crisis and pandemic plan as required. Explicit consideration on how lessons from previous and future exercises and AAR can contribute to strengthening this plan can ideally lead to strengthened preparedness and response capacities in the region. More detailed reporting and regular assessments of preparedness capacity at national level may increase accountability and highlight persisting gaps. Article 7 describes a triennial reporting which is “based on agreed common indicators”, thus a form of monitoring of progress similar to the SPAR. In addition to capturing pre-defined indicators through the templates that will be defined under this Article 7, efforts should also be made to encourage member states to address gaps identified through SimEx and AAR. Article 8 calls for regular “assessments of prevention, preparedness and response planning”, which can be read as evaluations complementing the monitoring described in Article 7. But Article 8 does not specify how these should be done – it is here that SimEx would be relevant to include as assessments. Simultaneously, AAR from the period in question should be taken into account. The Article describes a nine month delay to execute an action plan addressing the proposed recommendations, but only for Member States. The Commission should also consider how the results of multi-country exercises are followed up to address *overarching* issues which are not solved at State level, and how this should be monitored at a *regional* level.

### Limitations to this study

The review was conducted as a desk exercise and is based on the information found in the reports. We do not have secondary sources of information to triangulate our findings. The timeline of the included SimEx/AAR is long (2005–2018), during which time preparedness within the EU has been also evolving and developing. Numerous EU agencies, organisations, tools, SOPs and guidance documents have been produced or changed which are difficult to capture and contextualise from the outside. To mitigate this, we have invited key persons in the EC and other EU agencies to review the draft report, and have presented and discussed findings with peers in the Joint Action and members of the EC.

Only SimEx and AAR organized by the EC were included in this analysis, which means only a small number of exercises were reviewed. Exercises at national level have been carried out in the same time period, and might produce other types of recommendations. The scope and topics of the exercises and selection of participants. Selecting common events or rare events as exercise scenarios can influence the result as some participants may be more familiar with scenarios that occur relatively frequently than those who do not, which might influence the nature and quality of recommendations and which IHR core capacities addressed. There will be a certain bias in which capacities are addressed in an exercise because some capacities lend themselves better than others to assessment in an exercise setting.

Finally, this report provides a comparative analysis of data from different sources and different times that were not specifically designed to be compared. Recommendations from SimEx and AAR are not conceived according to the IHR core capacities, and there is therefore some subjectivity in the interpretation of the recommendations and classification according to IHR (2005) capacities.

## Conclusions

This study has assessed the added value of using results from SimEx and AAR in assessing preparedness and response at regional level in the EU. It finds that these qualitative tools provide valuable insight in the state of public health preparedness at EU level and are therefore highly useful evaluation tools. The recommendations from the SimEx and AAR from the period in this report were found to be highly relevant in identifying important gaps in health preparedness in the EU, particularly in the light of the lessons learned from Covid-19. This finding underline how many cross-national preparedness gaps exposed during the COVID-19 pandemic had long been known, and that a better follow-up and implementation of recommendations from SimEx and AAR could have improved the response.

At the regional level there are few other mechanisms in place to assess IHR (2005) compliance. SimEx and AAR are valuable instruments to assess the functionality of preparedness and response mechanisms as well as to train and raise awareness on health emergencies. They should be regarded as complementary to existing IHR monitoring tools at national level such and SPAR and JEE, and possibly other monitoring tools at regional level in the EU. With the new European Health Union, the EU will have an even more important role in coordinating health emergencies and evaluating the capacity at EU level will become more important in the future. However, implementation of recommendations remains a challenge and is an important barrier for maximising the value of SimEx and AAR in improving public health preparedness in the EU.

Based on our conclusions, we recommend that regional SimEx should be carried out following [15] Guideline for SimEx and AAR, as an opportunity both to evaluate functionality and to practise response mechanisms, and that AAR should be carried out routinely after public health events. SimEx should be designed to target key gaps and challenges for regional preparedness, based on previously identified issues both from monitoring tools, risk analyses and previous evaluations. Better systems for follow up and implementation of recommendations is crucial to have an impact on the EU’s ability to respond effectively to public health crises. To improve implementation, the results from SimEx and AAR needs to be transferred to relevant authority in an effective way and a plan for follow up should be developed after each exercise, including assigned responsibilities and a plan for accountability. Organisers of regional SimEx should centralise information about the implementation of recommendations, to enable identifying the areas where progress have been done and not. Lastly, further investigations of the existing barriers for progress in implementation is warranted to target further measures for improving public health preparedness in the EU. 

### Supplementary Information

**Additional file 1. Annex 1.** Comparison between recommendations to states and SPAR indicators. **Annex 2. **Recommendations to EU agencies in reports reviewed. **Annex 3.** Comparison of recommendations with lessons learnt from COVID-19.

## Data Availability

All data analysed in the study is available from the CircaBC repository, https://circabc.europa.eu/ui/welcome for users with access, or from the author upon reasonable request. The dataset generated during the study is available from the author upon reasonable request.

## Abbreviations

AAR: After Action Reviews
CHAFEA: Consumers, Health, Agriculture and Food Executive Agency (executive EU agency)
DG ECHO: Directorate-General for European Civil Protection and Humanitarian Aid Operations
DG HOME: Directorate-General for Migration and Home Affairs
DG SANTE: Directorate-General for Health and Food Safety
ECDC: European Centre for Disease Prevention and Control (Decentralised EU agency)
EEA: European Economic Area
EFSA: European Food Safety Authority (Decentralised EU agency)
EMA: European Medicines Agency (EMEA) (Decentralised EU agency)
HERA: European Health Emergency preparedness and Response Authority
IAA: Intra Action Review
IHR (2005): International Health Regulations
IPCEI: Important Project of Common European Interest
JEE: Joint External Evaluation
JRC: Joint Research Centre (European Commission's science and knowledge service)
M&E: Monitoring and Evaluation
MEF: Monitoring and Evaluation Framework
MS: EU Member State
SimEx: Simulation Exercises
JA-SHARP: Joint Action on Strengthened International HeAlth Regulations and Preparedness in the EU
SOP: Standard Operating Procedure
SPAR: State Party self-assessment annual reporting

## References

[1] Carlson CJ, Albery GF, Merow C, Trisos CH, Zipfel CM, Eskew EA, Olival KJ, Ross N, Bansal S (2022). Climate change increases cross-species viral transmission risk. Nature.

[2] Bartolini G (2021). The failure of ‘Core Capacities’ under the Who International Health Regulations. ICLQ.

[3] Haider N (2020). The Global Health Security index and Joint External Evaluation score for health preparedness are not correlated with countries' COVID-19 detection response time and mortality outcome. Epidemiol Infect.

[4] Kandel N, Chungong S, Omaar A, Xing J (2020). Health security capacities in the context of COVID-19 outbreak: an analysis of International Health Regulations annual report data from 182 countries. Lancet.

[5] WHO (2022). Strategic Partnership for Health Security and Emergency Preparedness (SPH) Portal.

[6] WHO (2022). Strategic Partnership for Health Security and Emergency Preparedness (SPH) Portal.

[7] R. Shiny from R studio. 2022. Available: https://shiny.rstudio.com/. Accessed Oct 2022.

[8] European Commission. Drawing the early lessons from the COVID-19 pandemic. Communication from the commission to the European parliament, the European council, the council, the European economic and social committee and the committee of the regions. Brussels: European Commission; 2021.

[9] Copper FA, Mayigane LN, Pei Y, Charles D, Nguyen TN, Vente C, Chiu De Vázquez C, Bell A, Njenge HK, Kandel N, HO ZJM, Omaar A, De La Rocque S, Chungong S (2020). Simulation exercises and after action reviews - analysis of outputs during 2016–2019 to strengthen global health emergency preparedness and response. Global Health.

[10] Stoto MA, Nelson C, Piltch-Loeb R, Mayigane LN, Copper F, Chungong S (2019). Getting the most from after action reviews to improve global health security. Glob Health.

[11] European Centre For Disease Prevention and Control (2023). Lessons from the COVID-19 pandemic.

[12] Parker GW (2020). Best practices for after-action review: turning lessons observed into lessons learned for preparedness policy. Rev Sci Tech.

[13] WHO. Report of the Global consultation on after action reviews and simulation exercises under the IHR monitoring and evaluation framework. Tunis: World Health Organisation; 2019.

[14] WHO. Reflect, Adjust and Improve. Emergency Preparedness and Response during a Pandemic. A global analysis og COVID-19 intra-action reviews. 2022a. Unpublished manuscript.

[15] WHO (2018). Country implementation guidance: after action reviews and simulation exercises under the International Health Regulations 2005 monitoring and evaluation framework (IHR MEF).

[16] WHO (2020). Guidance for conducting a country COVID-19 intra-action review (IAR).

[17] Anagnostopoulos L, Kourentis L, DávilaCornejo M, Lorente IM, Dionisio M, Marotta C, Hadjichristodoulou C, Mouchtouri VA (2022). Using the Intra-Action Review Methodology at European Level to Assess Effectiveness of Measures for Cruise Ship Operations in the COVID-19 Context. Med Sci Forum.

[18] Boland M, Morrissey MC, O'connor E, Dever N, O'mahony C, Romanovski S, O'riordan M (2022). Intra-Action Review of the HSE Health Protection response to the COVID-19 pandemic during 2021: Final Report and Recommendations. Health Service Executive.

[19] O’connor E, O’riordan M, Morrissey MC, Dever N, O’mahony C, Romanowski S, Boland M (2023). A methodological approach to intra-action reviews - application and adaptation of existing global guidance during the COVID-19 pandemic response in Ireland, 2021. Eurosurveillance.

[20] European Parliament (2022). Regulation (EU) 2022/2371 Of The European Parliament And Of The Council of 23 November 2022 on serious cross-border threats to health and repealing Decision No 1082/2013/EU.

[21] European Commission (2020). Building a European Health Union: Reinforcing the EU’s resilience for cross-border health threats. Communication from the commission to the European parliament, the council, the European economic and social committee and the committee of the regions.

[22] European Centre For Disease Prevention And Contro (2017). Guide to revision of national pandemic influenza preparedness plans - Lessons learned from the 2009 A(H1N1) pandemic.

