# Supplementary material for: Assessing public health preparedness and response in the European Union- a review of regional simulation exercises and after action reviews
Source: Global Health. 2023 Oct 28;19:79. doi: 10.1186/s12992-023-00977-y (PMC10612297; doi:10.1186/s12992-023-00977-y)
Supplement: Supplementary file 1 — Additional file 1. Annex 1. Comparison between recommendations to states and SPAR indicators. Annex 2. Recommendations to EU agencies in reports reviewed. Annex 3. Comparison of recommendations with lessons learnt from COVID-19. [file 12992_2023_977_MOESM1_ESM.doc]

Annex 1: Comparison between recommendations to states and SPAR indicators

| **Recommendations to EU Member States/EEA/Switzerland** | **SPAR scores for EU/EEA/Switzerland** |
| --- | --- |
| **C1/C3: Policy, legal, financing**    7 recommendations from 4 different SimEx. They identify a need to clarify roles, responsibilities and mandates of different actors, as well as collaboration, through written agreements or SOPs. Three of them are about defining cross-border sharing and protection of information. One recommendation is about separating between political and technical meetings.    The recommendations indicate that necessary systems are in place but the awareness of these systems and how they function needs to be improved. This is an aspect that the SPAR does not cover, as it is more focused on identifying gaps and legally covering them. The recommendations thus provide detail and context in relation to the legal framework for IHR, and identify specific issues where SOPs are needed. | 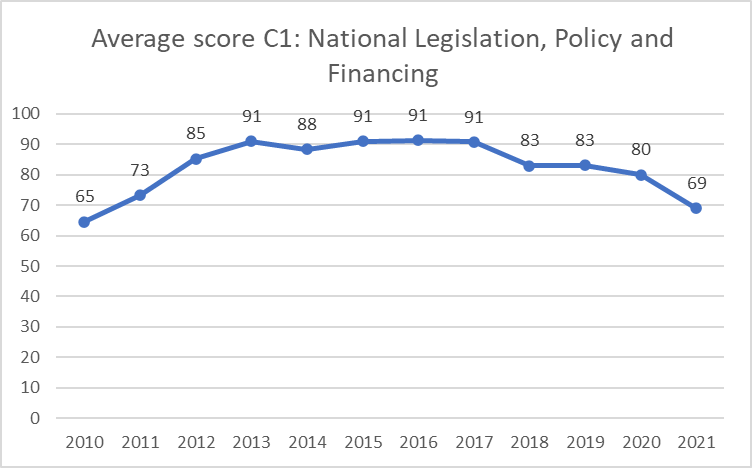 |
| **C2: IHR coordination and National IHR Focal Point**    31 recommendations from 12 SimEx.  The majority, 16, concern intersectoral or international communication, including the use of information exchange platforms such as HEDIS or EWRS. Mostly, it is about improving the awareness and use of the channels, and improving information exchange between sectors and countries. Many recommend reconsidering which actors have access to which channels. 7 of them pertain to encouraging cross-sectoral collaboration, by including different sectors in both preparedness and response to incidents. The remaining 8 are about how to clarify mandates of stakeholders, and improve the organisation and quality of responses to events.    SPAR scores cover multisectoral coordination and communication well, however do not assess international comms and collaboration. This is an added value of the recommendations.  Further, the recommendations identify specific gaps in the coordination, providing more details than the SPAR. | 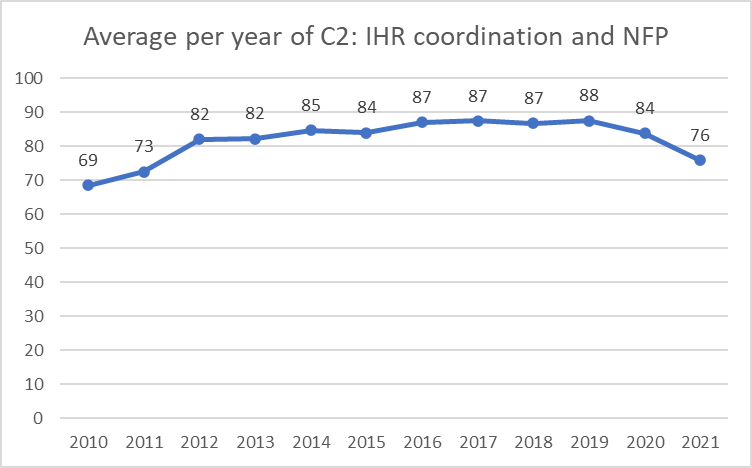 |
| **C4: Laboratory**    6 recommendations from 4 SimEx.  Better understanding of roles and responsibilities of natl/intl reference laboratories during crises, as well as sharing information about their capacities. Trainings on epidemic intelligence for labs. Agree international methodologies for strain comparison. Develop directory of carriers of dangerous goods. Establish procedures and advice to private laboratories about sample management of notifiable diseases.    SPAR indicators are focused on systems being in place for specimen referral and transport, as well as a biosafety and biosecurity regime. The recommendations indicate that information sharing and interoperability needs to improve, and identify specific areas of improvement, thus complementing SPAR. | 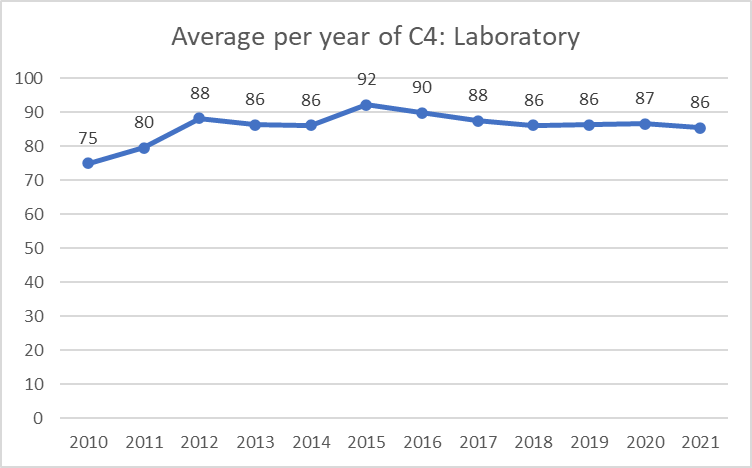 |
| **C5: Surveillance**    5 recommendations in 4 different exercises. They include recommendations on protocols relating to climate change related illness and vector control. Two of the recommendations concern the access to medical and epi data for other actors during events. One is about international coordination of contact tracing policies.    The SPAR scores are based on the existence of national surveillance systems and protocols to define them, whereas the recommendations focus also on the exchange of information between the surveillance system and other actors as well as internationally. The SimEx thereby covers an area of functionality that SPAR does not cover. | 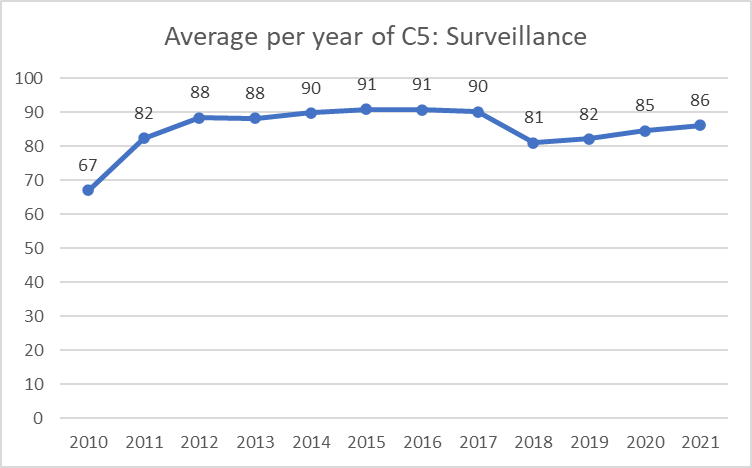 |
| **C6: Human resources**    10 recommendations in 7 different exercises.  Four of the recommendations concern ensuring an appropriate workforce during crises through preparatory trainings, in particular to ensure forensic epidemiologists. The remaining points recommend specific trainings, often for a wide group of stakeholders.    Whereas the SPAR details whether a workforce is available, most of the recommendations specify skills that need improvement or knowledge gaps in different workforces or in general. The SimEx is thereby a complement to the SPAR scores providing specific qualitative input. | 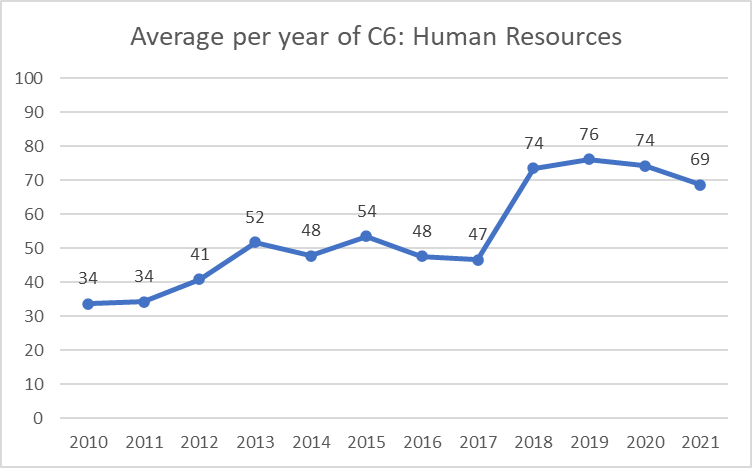 |
| **C7: Health emergency management**    29 recommendations in 13 exercises.  8 of them concern how to share information and communicate across sectors, countries and EU organisations. 7 are about sharing and streamlining practises and strategies, such as alert level definitions and response mechanisms. 4 pertain to the organisation and quality assurance of the EOC. 5 are about planning for specific types of emergencies. Three recommend writing or reviewing SOPs for cross-sectoral collaboration, and two pertain to vaccination during pandemics.    SPAR scores are focused on the presence of plans and management systems at the national level. The recommendations are more focused on the fact that health emergencies often involve several countries, highlighting the importance of information sharing and coherence between countries. | 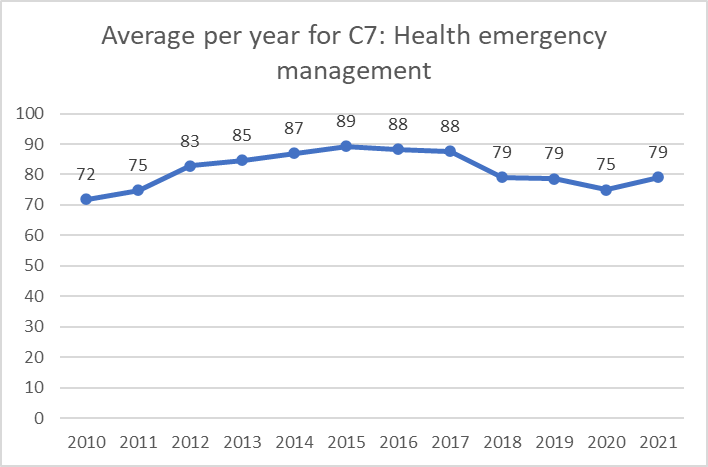 |
| **C8: Health services provision**    4 recommendations in 3 SimEx. Pertain to preparedness for cyber-attacks on hospitals, improving business continuity during pandemics in light of the impact it may have on health services, and management of antiviral drugs. | New capacity since 2018, scores have been 81 and 82 up to 2021. |
| **C10: Risk communication and community engagement**    23 recommendations from 10 SimEx.  8 of these are about organisation and strategy in communication work, including protocols and SOPs to develop/review, as well as intelligence gathering. 5 concern the specific communication tools used to reach the public or specific groups in order to have the best possible impact. A further 6 are about how to improve the quality of the content of messages, in particular how to ensure technical accuracy and coherence of messages across countries. 4 recommendations pertain to specific topics: climate change and anti-vaccine issues.    SPAR indicators for C10 are fairly detailed and include a variety of factors to take into account. In this sense, the recommendations do not provide much further value, as they are quite general and cover more or less the same issues as the SPAR indicators. | 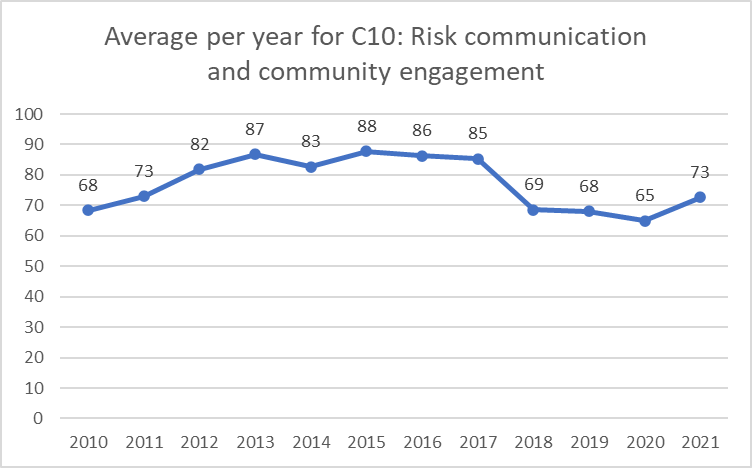 |
| **C11: Points of Entry**    One recommendation.  Member states should share information regarding travel advice and the Commission aim for a coordinated and common approach to travel information and restrictions across Europe. | 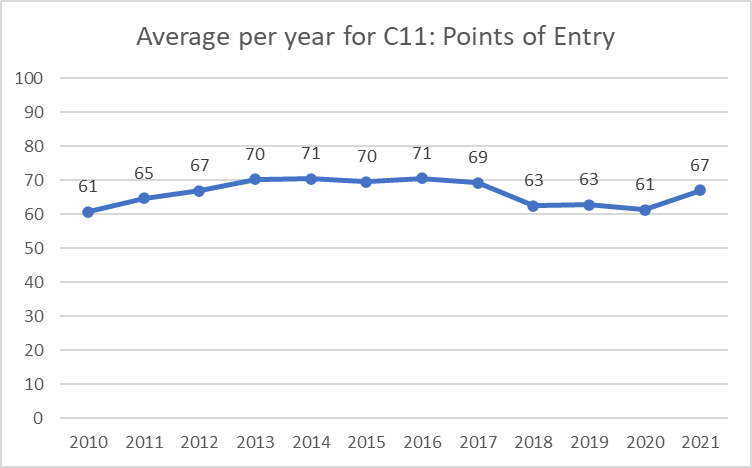 |
| **C12: Zoonotic diseases**    One recommendation: Consider how veterinary practices and human health practices in the private sector can be more engaged in the One Health approach at the national level, as envisaged under Animal Health Regulation (EU) 429/2016.  Other recommendations stress the importance of improving collaboration between the human and animal health sectors, but fall under other categories. | 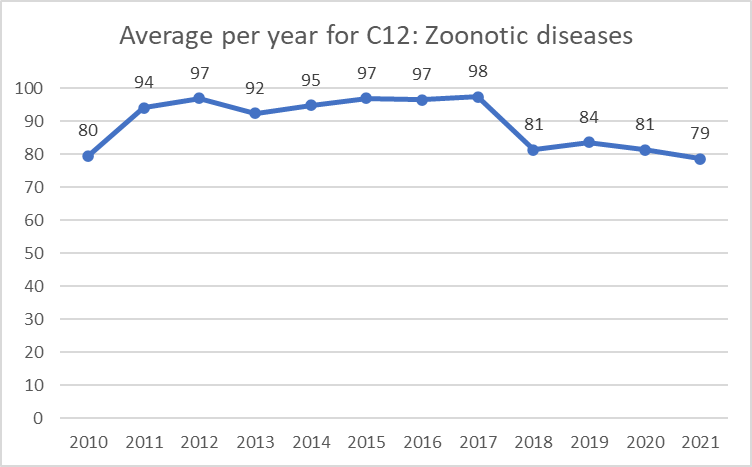 |
| **C 13: Food safety**    No recommendations given to states within this capacity. | 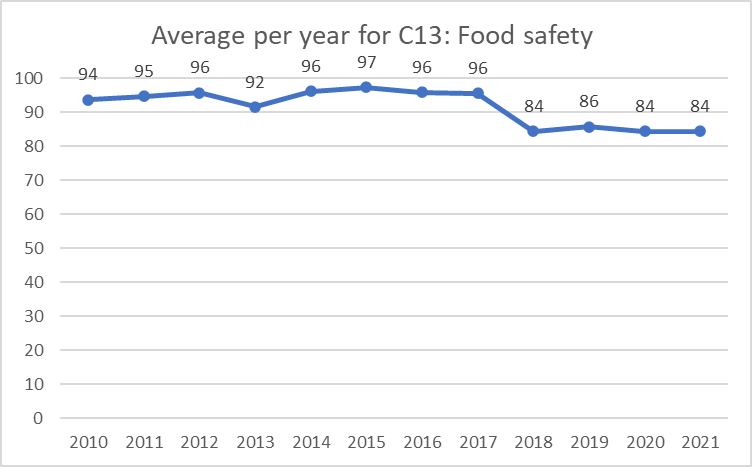 |
| **C14: Chemical events**    Three of the SimEx in the dataset had scenarios about chemical events. Most of the recommendations in the exercises however fall under other capacities.  Two recommendations from one SimEx recommend reviewing the capacity of poison centres to provide advice, and to facilitate access to notifications of chemical events in EWRS | 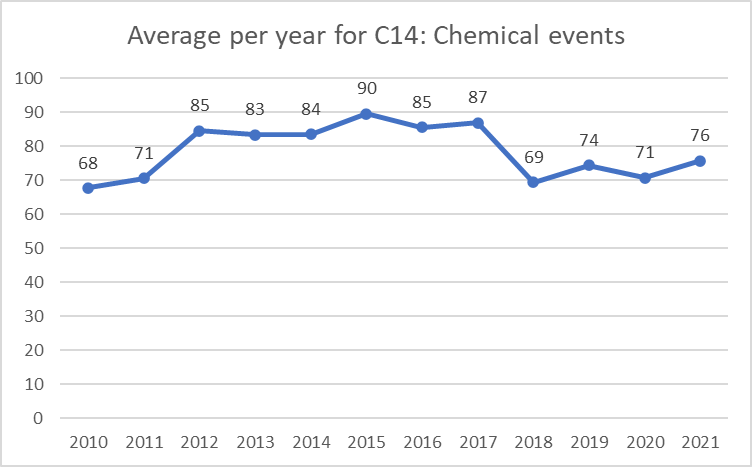 |
| **C 15: Radiation emergencies**    No recommendations given to states within this capacity. | 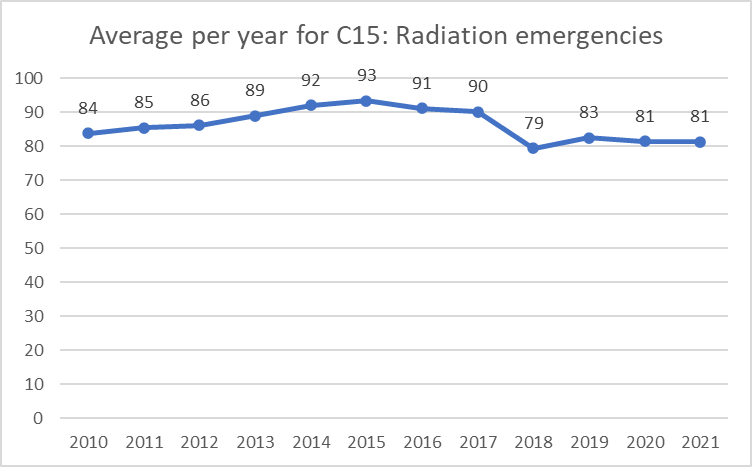 |

Annex 2 : Recommendations to EU agencies in reports reviewed

| **Main objective** | **Exercises** | **Summary of recommendations** |
| --- | --- | --- |
| Influenza preparedness | 2005  COMMON GROUND      2009  TOR 1 | 1- Develop a generic national plan for emergency frameworks  2- Investigate and clarify international regulations on travel and border control during international infectious disease outbreaks – aim for a coordinated approach across EU MS.  3- Review, define, improve and train on communications tools for both epi data and business continuity (**HEDIS**, **EWRS, MedISys** and others)  4- Establish secured digital platform for EU communication  5- Engage with pharma and vaccine manufacturers to ensure access to MCM for the EU during public health emergencies  6- Clarifications of roles of ECDC, EC, WHO and WHO EURO  7- Streamline key EC-wide media responses  8- Enhance coordination and reporting mechanisms between WHO, EU agencies and MS.  9- Develop a strategy for HSC COMNET with clear roles, responsibility, line of communication, trigger points and coordination mechanism |
| Vaccination during an infectious disease emergency | VACCINE WORKSHOP  (2009)  TOR 2  (2010) | 1- Develop an EU joint procurement protocol which ensures equitable distribution between MS and addresses national requirements, logistics and legal issues  2- Develop a regional stockpiling mechanism managed by EU or WHO.  3- Enhance collaboration and coordination between DG SANCO, EMEA, ECDC, HSC communicators and WHO |
| Communication during a biological health threat of an infectious agent. | 2005  NEW WATCHMAN | 1- Develop SOPs in case of public health emergency with defined roles and responsibilities of MS and ECDC, EC, WHO and WHO EURO.  2- Establish minimum standard of equipment needed for teleconferences during crises  3- Develop secure system for sharing information across EU   4- EC to ensure streamlined alert level definitions across MS  5- Ensure coordinated key EU wide responses to media  6- Conduct routine exercise to enhance coordination between MS and ensure that identified issues are addressed |
|  | 2008  UNITED HORIZON | DC SANCO should upgrade the **HEDIS** system by:  1- End user survey to understand needs and requirements    2- SOPs, guidance document and training on how to use HEDIS    4- Clarify role of HEDIS in relation to EWRS and other EU and WHO systems    5- Address technical and security issues   6- Develop a mechanism for instant sharing of urgent information |
| Communication in addressing a radio-nuclear threat. | 2011  AQUA UTOPIA | **HSC and HSC COMNET should**  1- Develop and update TORs governing the mandates of HSC and HSC COMNET  2- Reinforce knowledge internally of mandates and procedures  3- Define triggers, actions and joint criteria for information sharing during crises  4- Receive training on use of HEDIS  5- Improve overview and access to specialist advice, including on CBRN threats  6- Develop use of social media as a tool  7- Map communication processes in MS and share best practises |
| Communication in mitigating a chemical event. | 2011  IRIDIUM 1 | **DG SANCO should:**  1- Use updated contact list and e-mail protocol for incident notification  2- Define a process for developing a case definition of chemical incidents  3- Rapidly provide and share an overarching picture of incidents with EU and MS  4-Coordinate scientific advice for chemical incidents with MS to avoid conflicting advice  5- Consider agreements on rapid collaboration and info sharing in border regions  6- Ensure involvement of both health and other sectors in preparedness planning  7- Review mutual aid plans between EU countries to streamline processes and ensure rapid deployment of assistance to neighbouring countries  8- Define protocols for RAS-CHEM  9- Consider joint public messaging communications |
| Intersectoral coordination in addressing biological threat with a direct impact on food safety. | 2008  AEOLUS | 1- Establish a European coordination mechanism for health events that are not due to a communicable disease, and define which European scientific body would lead on risk assessment and epidemiological investigation  2- Review protocols for exchange of information during health threats of criminal/terrorist nature, and establish guidance on management, access and transfer of sensitive data during such crises  3- Establish an EU-wide timeline for audio-conferences, reporting, risk assessments and communications - HEDIS provides a Common Recognized Information Picture.  4- DG SANCO and HSC COMNET should ensure consistency of public messages by establishing a framework for sharing information about public statements.  5- Develop a policy for EU information systems HEDIS, EWRS, RAS-BICHAT        - Define function, usage and interplay between systems, and who has access         - Review and improve user friendliness, security and resilience of the systems         - Establish verification of shared information. |
|  | 2013  ARISTAEUS | **DG SANCO should:**  1- Develop EU-wide SOPs on emergency management of food-borne outbreaks, defining roles and responsibilities, coordination mechanisms, lines and tools of communication  2- Secured digital cross-sectoral platform for cross communication between MS and relevant EU agencies to share information, actions to take, epi and lab data.  3- Secured digital platform for public dissemination    4- A protocol for coordination enables exchange of expertise between HSC members and HSC CommNet members.  5- Define EFSA’s mandate and develop SOPs for joint ECDC/EFSA food borne outbreaks  6- Review needs and access to technical assistance and training on outbreak response |
| Intersectoral coordination during a radio-nuclear event | 2010  ECLIPSE | 1- Develop a European 'uncertainty’ communication strategy for the early stages of an incident with generic messages to be used  2- Study, monitor and utilise social media platforms for community engagement  3- Defined trigger points for inclusion of technical expertise   4- Ensure awareness and training for senior officials |
| Intersectoral coordination in facing a biological threat of zoonotic nature. | 2017  ALPHA | 1- Multisectoral coordination protocol between the EU commission, and OIE on emerging diseases in animals which defines     - Multisectoral coordination between animal and human health authorities     - Roles and responsibilities of relevant EU agencies and lines of communication.   C- SOPs for surveillance data on emerging diseases, and AMR.  3- Development of joint dashboard or information sharing system for zoonotic threats.  4- Increased awareness and training on the One Health interface. |
| Intersectoral coordination in mitigating a hybrid threat. | 2018  Chimera | 1- Develop common EU guidelines on hybrid threats  2- A secure system for exchanging information in case of hybrid threats.  4- An emergency management protocol for hybrid threats including roles, responsibilities, coordination mechanism, line and mode of communication, and contingency plan.   5- Address the need to communicate and coordinate with non-EU states |
| Cross-border coordination in mitigating a chemical event. | 2014  Quicksilver | 1- Develop and strengthen the HEOF  2- Improved process for the timely sharing of information and use of tools such as EWRS and RASCHEM between the relevant institutions at the EU and MS to ensure rapid risk assessment and crisis communication  3- Continued development of the HSC Communicator’s Network and awareness of its role and responsibilities, to ensure coherent messaging at European level.  4- Implement procedures for sharing technical information between EU agencies and WHO to ensure unified and scientifically based advice to MS |
|  | 2015  Quicksilver Plus | 1- DG SANTE to develop its role as a European coordinator during cross border threats   2- Improve awareness of roles, responsibilities and communication mechanisms between EU agencies, WHO, MS.  3- Establish SOPs between DG SANTE, DG ECHO, DG HOME.  4- Upgrade and train on functionality of RASCHEM and EWRS.  5- Review and increase awareness of HEOF roles and responsibilities  6- Develop HSC COMNET and a unified communication platform |
| Intersectoral coordination in a biological threat | 2018  TARANIS | 1- Improve business continuity planning, resilience, intersectoral and cross-border cooperation during disruptive events  2- Improve collaboration between public health and other critical sectors for the maintenance of public services |
| Preparedness and response facing biological threats due to climate change | 2016  Orion | 1- Improve preparedness in terms of response coordination, scientific evidence of vector control measures, medical countermeasures, communication and public health adaptation to climate change  2- Develop European guidelines on how to disinfect airplanes in case of PHE  3- Develop a European universal communication tool for all hazards   4- Enhance intersectoral collaboration by conducting further exercises and trainings |
| Laboratory capacities during a biological threat of an infectious agent. | 2011  HERMES | **Review the legislative framework for transport of dangerous goods between EU MS**  1- Define roles and responsibilities between MS, EU agencies, IATA and others.  2- Establish a list of EU accredited 'Expert labs'  4- Review border restrictions to enable movement of biological samples between MS  5- Establish standardized protocol on labs biosafety and biosecurity on packaging and transportation of infectious substances,   6- Implement online trainings and accreditation. |

*Annex 3: Comparison of recommendations with lessons learnt from COVID-19*

| *Covid-19 lesson 1:* ***“Faster detection and response depends on stronger global surveillance and more comparable and complete data.”***    The report highlights that current international alert system did not operate with sufficient speed and recommends implementation of a new global surveillance system and a new and improved European pandemic information gathering system. | | |
| --- | --- | --- |
| **SimEx/IAR**  **(type of event)** | **Year** | **Similar recommendations** |
| COMMON GROUND  Influenza preparedness | 2005 | Established that efficient on-line, real-time data input and access by the relevant bodies is needed in a crisis situation and would alleviate the duplication of case reporting. |
| TOR 1  Influenza preparedness | 2009 | Review and define the use of EWRS, HEDIS, and MedISys. A review of reporting processes should be undertaken by ECDC and WHO Euro *of the case reporting system and other similar systems to ensure an efficient process is developed for use between multiple receiving agencies.* |
| ARISTAEUS  biological threat (food safety) | 2013 | Identified the need to organise central collection, collation, analysis and dissemination of data and information during multi-state outbreaks, facilitated by timely provision of data from Member States. |
| ALPHA  biological threat  (zoonotic) | 2017 | *The Commission should implement the interactive information system referred to within Animal Health Regulation (EU) 2016/429.*    *The Commission should develop procedures to support intersectoral sharing of information on emerging diseases when the disease is not notifiable.* |
| TARANIS  biological threat (pandemic flu) | 2018 | *Strengthen the role of information sharing, risk identification and surveillance between EU agencies, in particular ECDC, and national / EU / international partners.* |
| Covid-19 lesson 2: ***“Clear and coordinated scientific advice facilitates policy decisions and public communication.”***    More coordination at EU level on scientific advice was identified in the report as a need to ensure consistent, coherent and factual communication. A new European Chief Epidemiologist was proposed to address this. | | |
| **SimEx/IAR**  **(type of event)** | **Year** | **Similar recommendations** |
| IRIDIUM  Chemical event | 2011 | MS and DG SANCO should coordinate the provision of scientific advice for response to chemical incidents to ensure that conflicting advice is not given. |
| Quicksilver  Chemical event | 2014 | Series of recommendations on how to ensure coherent public messaging from MS and the EU. For example:   - EU and MS should coordinate crisis communications - Communicators and scientific staff should collaborate on communications, and to share public information before releasing it. - *MS should consider the advice of the EU Scientific Committee in the context of cross-border health threats as the one to be used (coherence of messages and increased safety for the public)* |
| Orion Biological treat (chikungunya virus outbreak) | 2016 | - *Develop a protocol to enable daily key messages to be released to MS to aid coordination including intersectoral during an incident* - *Implement a system to facilitate interaction and sharing of information to inform decision-making between MS* |
| Covid-19 lesson 3: ***“Preparedness needs constant investment, scrutiny and review”***    It was concluded in the report that even if there were many preparedness plans, there was a lack of systems and means to put these into action. Need for stepping up investment was acknowledged, and an annual State of Preparedness Report was suggested. | | |
| **SimEx/IAR**  **(type of event)** | **Year** | **Similar recommendations** |
| TOR 1  Influenza preparedness | 2009 | - MS, the Commission and EU Agencies *should continue to evaluate pandemic preparedness for sectors and services identified as potentially at risk, (health and cross-sectoral), particularly as not all sectors experienced similar levels of pressure.* - MS and EU Agencies should *undertake a pandemic “lessons identified” review process and the outcomes, where appropriate, are shared across MS and the EU. Outputs of this process should be inputted into the development of pandemic plans at MS and EU level.* - The Commission and EU Agencies should *refine and publicise estimates of pandemic planning assumptions for a new pandemic as early as possible to enable other sectors to prepare and ensure these are reviewed as the pandemic progresses.* |
| Quicksilver,  Quicksilver Plus, Chimera  Chemical event/Hybrid threat | 2014 2015 2018 | Training including at Member States and/or EU level was mentioned in three exercises, including *continual programme or regular training and exercises.* |
| Covid-19 lesson 5*:* ***“Coordinated measures should become a reflex for Europe”***    The report highlights further strengthening of inter-institutional cooperation, coordinate decision and combine necessary expertise and propose building of the European Health Union. | | |
| **SimEx/IAR**  **(Type of event)** | **Year** | **Similar recommendations** |
| COMMON GROUND  Influenza preparedness | 2005 | Clarifying the roles of organisations such as ECDC, EC WHO Europe and WHO HQ so that Member States could make coordinated and informed decisions concerning, for example, the movement of individuals. |
| TOR1  Influenza preparedness | 2009 | Share information regarding travel advice and have a coordinated approach and share contact tracing policies. |
| Quicksilver Plus chemical event | 2015 | Recommended that *DG SANTE to consider including roles and responsibilities of partner organisations within HEOF SOPs and emergency plans.* |
| TARANIS  biological threat (pandemic flu) | 2018 | Recommended to *Clarify roles and responsibilities of all stakeholders during the cross border intersectoral response to disruption during a pandemic.* |
| Covid-19 lesson 6: ***“Reinforced public-private partnerships and stronger supply chains are needed for critical equipment and medicines”***    The need to increase resilience of supply chain for critical supplies was addressed in the report. Joint procurements and stockpiles were mentioned as some of the measures EU has taken during the pandemic. The establishment of HERA to ensure preparedness and response in terms of medical countermeasures, and IPCEI to enable breakthrough innovation in pharmaceuticals were advised. | | |
| **SimEx/IAR**  **(type of event)** | **Year** | **Similar recommendations** |
| COMMON GROUND  Influenza preparedness | 2005 | Highlighted the need to engage with vaccine manufacturers and pharmaceutical companies to resolve “issues of availability and suitability of containment measures”, specifically development and procurement of vaccines, the quantities required and the projected timeframes, as well as co-ordinated distribution and use of anti-virals and vaccines, and mechanisms to allocate vaccines and anti-virals between Member States. |
| TOR1  Influenza preparedness | 2009 | Recommended that MS incorporate planning for the provision of mutual aid as part of generic business continuity planning for health services, including health sector supply and support services. |
| TOR 2 influenza vaccination | 2010 | Joint procurement enables stronger negotiation power, lower costs, and equitable access, and that these JP should adapt to national requirements, logistics, context and legality. |
| TARANIS  biological threat (pandemic flu) | 2018 | Identified the critical reliance on supply chains and the need to have contingency plans in cases of disruptions, where all countries would be demanding similar limited resources, including medical countermeasures and personal protective equipment. The loss of such services could quickly make the provision of the effective response unsustainable. A thorough review into the resilience of key supply provision was recommended as well as more coordination and guidance on EU level. |
| Covid-19 lesson 7: ***“A pan-European approach is essential to make clinical research faster, broader and more effective”***    The report states that the approach to clinical trials in Europe was fragmented and divided in the beginning of the pandemic. An EU wide trial network has been supported by the Commission and a large-scale EU platform for multi-centre trials is recommended to be set up. | | |
| **SimEx/IAR** | **Year** | **Similar recommendations** |
| TOR 2  influenza vaccination | 2010 | The IAR on vaccine strategies established *need for public clinical research capacity (e.g., carry out comparative effectiveness studies) in the EU, (*…) *this should be coordinated by an existing EU agency.* |
| Covid-19 lesson 8: ***“Capacity to cope in a pandemic depends on continuous and increased investment in health systems”***    Structural weakness in health systems, like lack of surge capacity, and the need for health systems in Europe to become more resilient was concluded in the report. It is advised to support MS in this process. | | |
| **SimEx/IAR** | **Year** | **Similar recommendations** |
| TOR 1 Influenza preparedness | 2009 | *Appropriate that MS incorporate planning for the provision of mutual aid as part of generic business continuity planning for health services, including health sector supply and support services.* |
| *TOR 2 Influenza vaccination* | 2010 | *Better coverage of health care professionals is essential to maintaining health care services in a pandemic. Low coverage is also an obstacle to reaching target/risk groups as well as the general public.* |
| TARANIS biological threat (pandemic flu) | 2018 | Frontline and support staff shortages in emergency and community-based care, e.g., in a pandemic situation, would severely hamper capability and capacity. Recommend considering the impact on the health sector and to identify and prioritise services to assign staff to critical services. |
| Covid-19 lesson 10: ***“A more coordinated and sophisticated approach to tackling misinformation and disinformation should be developed”***    Misinformation and disinformation have been spread with great speed and the report highlights the need for standby capacity to combat these risks and recommends improved tools for better coordination to detect and react at EU level. | | |
| **SimEx/IAR** | **Year** | **Similar recommendations** |
| VACCINE WORKSHOP  influenza vaccination | 2009 | Raise the issue of how to counter the “anti-vaccine lobby” and recommending that scientific evidence will be important in this. |
| TOR 1 Influenza preparedness | 2009 | Considered important that MS monitor the accuracy of public health messages issued by the media during a crisis and develop systems that assess the level of public understanding of the issued messages. |
| TOR 2  influenza vaccination | 2010 | In relation to vaccination strategy during a pandemic, the recommendations were among others:   - to tackle any vaccination fears and or criticisms ‘head on’ through an open debate - use specialised communication for at risk groups - consider targeted communications - use social media, newspaper and mass media sites to gain information of antivaccination campaigns. |
| ALPHA biological threat (zoonotic) | 2017 | Active role for risk communicator was highlighted *Future exercises should include an active role for communicators from Animal Health and Public Health services to clarify or test the requirements of risk and crisis communication.* |

ANAGNOSTOPOULOS, L., KOURENTIS, L., DÁVILA CORNEJO, M., LORENTE, I. M., DIONISIO, M., MAROTTA, C., HADJICHRISTODOULOU, C. & MOUCHTOURI, V. A. 2022. Using the Intra-Action Review Methodology at European Level to Assess Effectiveness of Measures for Cruise Ship Operations in the COVID-19 Context. *Medical Sciences Forum,* 13**,** 14.

BOLAND, M., MORRISSEY, M. C., O'CONNOR, E., DEVER, N., O'MAHONY, C., ROMANOVSKI, S. & O'RIORDAN, M. 2022. Intra-Action Review of the HSE Health Protection response to the COVID-19 pandemic during 2021: Final Report and Recommendations. Health Service Executive.

EUROPEAN CENTRE FOR DISEASE PREVENTION AND CONTROL 2023. Lessons from the COVID-19 pandemic. Stockholm: ECDC.

O’CONNOR, E., O’RIORDAN, M., MORRISSEY, M. C., DEVER, N., O’MAHONY, C., ROMANOWSKI, S. & BOLAND, M. 2023. A methodological approach to intra-action reviews - application and adaptation of existing global guidance during the COVID-19 pandemic response in Ireland, 2021. *Eurosurveillance,* 28**,** 2200475.

PARKER, G. W. 2020. Best practices for after-action review: turning lessons observed into lessons learned for preparedness policy. *Rev Sci Tech,* 39**,** 579-590.

STOTO, M. A., NELSON, C., PILTCH-LOEB, R., MAYIGANE, L. N., COPPER, F. & CHUNGONG, S. 2019. Getting the most from after action reviews to improve global health security. *Globalization and Health,* 15**,** 58.

WHO 2020. Guidance for conducting a country COVID-19 intra-action review (IAR).
